# Knock down analysis reveals critical phases for specific *oskar* noncoding RNA functions during *Drosophila* oogenesis

**DOI:** 10.1093/g3journal/jkab340

**Published:** 2021-09-29

**Authors:** Andrew Kenny, Miles B Morgan, Sabine Mohr, Paul M Macdonald

**Affiliations:** Department of Molecular Biosciences, The University of Texas at Austin, Austin, TX 78712, USA

**Keywords:** noncoding RNA, *oskar*, karyosome, MTOC, oogenesis, shRNA

## Abstract

The *oskar* transcript, acting as a noncoding RNA, contributes to a diverse set of pathways in the *Drosophila* ovary, including karyosome formation, positioning of the microtubule organizing center (MTOC), integrity of certain ribonucleoprotein particles, control of nurse cell divisions, restriction of several proteins to the germline, and progression through oogenesis. How *oskar* mRNA acts to perform these functions remains unclear. Here, we use a knock down approach to identify the critical phases when *oskar* is required for three of these functions. The existing transgenic shRNA for removal of *oskar* mRNA in the germline targets a sequence overlapping a regulatory site bound by Bruno1 protein to confer translational repression, and was ineffective during oogenesis. Novel transgenic shRNAs targeting other sites were effective at strongly reducing *oskar* mRNA levels and reproducing phenotypes associated with the absence of the mRNA. Using GAL4 drivers active at different developmental stages of oogenesis, we found that early loss of *oskar* mRNA reproduced defects in karyosome formation and positioning of the MTOC, but not arrest of oogenesis. Loss of *oskar* mRNA at later stages was required to prevent progression through oogenesis. The noncoding function of *oskar* mRNA is thus required for more than a single event.

## Introduction

*Drosophila oskar* (*osk*) mRNA is required, independent of Osk protein, during oogenesis (Jenny *et al.* 2006). Mutants in which *osk* mRNA levels are substantially reduced or eliminated exhibit myriad defects. Most conspicuous of these is female sterility, a consequence of arrested oogenesis. Even before the arrest, more subtle defects appear: egg chambers often have too many nurse cells, the condensation of oocyte chromosomes to form the karyosome is incomplete, positioning of the microtubule organizing center (MTOC) within the oocyte is abnormal, certain ribonucleoprotein particles are disorganized, and proteins normally restricted to the germline cells of the ovary can be detected at low levels in surrounding somatic follicle cells (Jenny *et al.* 2006; Kanke *et al.* 2015; Kenny *et al.* 2021). Discovery of additional defects seems likely, awaiting only the application of appropriate assays. The diversity of *osk* RNA null mutant phenotypes, with no discernible shared dependence on a particular cellular pathway downstream of *osk* itself, might suggest the existence of multiple different noncoding functions. However, each phenotype listed above is sensitive to mutation of the same defined functional sequences in the *osk* mRNA 3’ UTR: a tightly clustered combination of Bruno1 (Bru1) binding sites, the *osk* noncoding element (ONCE), and A-rich sequences (ARS) (Vazquez-Pianzola *et al.* 2011; Kanke *et al.* 2015; Kenny *et al.* 2021). Thus, these elements either mediate multiple events or interactions, or they perform a single function with multiple pathways affected by disruption of that event. An extreme version of the latter option would be an initial “gateway” event with an immediate outcome, such as karyosome formation or proper MTOC organization, being a prerequisite for later outcomes, such as progression through oogenesis.

One approach to explore the relationship of different *osk* RNA null defects is to disrupt *osk* ncRNA activity at different stages of oogenesis, and ask if specific defects can be induced separate from others. Classical application of this strategy has often relied on temperature-sensitive mutants (*e.g.*, Jarvik and Botstein 1973). A more recent take on this concept relies on inducible gene activation or silencing *(e.g.*, Gossen and Bujard 1992; Gossen *et al.* 1995; Kumar *et al.* 2009; Fenno *et al.* 2011). In *Drosophila*, a useful method is gene knockdowns (KDs), relying on transgenic shRNAs under UAS/GAL4 control (https://fgr.hms.harvard.edu/fly-in-vivo-rnai) and a variety of GAL4 drivers (Brand and Perrimon 1993) with different temporal and/or spatial patterns of expression.

Here, we have taken the KD approach, commencing with the development of effective *osk*-shRNA transgenes to address a disabling limitation of the existing versions. We provide evidence of different critical periods for different *osk* ncRNA roles, and show that initial defects from early loss of *osk* mRNA do not have lasting effects if *osk* mRNA is restored.

## Materials and methods

### Flies and transgenes

To construct *osk-shRNA* transgenes, pairs of oligonucleotides were annealed and cloned into the *Nhe*I and *Eco*RI sites of the VALIUM22 vector. Oligonucleotide pairs, all shown with 5’ to the left: *osk-shRNA#1*, CTAGCAGTACGATTCTCTGCTGACGATTATAGTTATATTCAAGCATATAATCGTCAGCAGAGAATCGTGCG and AATTCGCACGATTCTCTGCTGACGATTATATGCTTGAATATAACTATAATCGTCAGCAGAGAATCGTACTG; *osk-shRNA#2*, CTAGCAGTAAGGAGATGCACAATATGCGATAGTTATATTCAAGCATATCGCATATTGTGCATCTCCTTGCG and AATTCGCAAGGAGATGCACAATATGCGATATGCTTGAATATAACTATCGCATATTGTGCATCTCCTTACTg; *osk-shRNA#3*, CTAGCAGTTCGACATACACTCCTGTCTAATAGTTATATTCAAGCATATTAGACAGGAGTGTATGTCGAGCG and AATTCGCTCGACATACACTCCTGTCTAATATGCTTGAATATAACTATTAGACAGGAGTGTATGTCGAACTG. The shRNA design was generously performed by Claire Yanhui Hu of the TRiP, Harvard Medical School. Each transgene was inserted at the attP40 site at 25C6 (Bloomington stock #25709).

The TRiP transgenic strain for KD of *osk* was TRiP.GL01101, Bloomington stock #36903, and for KD of *bru1* was TRiP.GL00314, Bloomington stock #35394.

The *osk^syn^* transgene was based on a rescuing genomic transgene (Kim-Ha *et al.* 1991) in which the portion of the *osk* gene from just 3’ to the start of the second exon to the stop codon (coordinates 8935984–8937367 in release r6.40 of the *D. melanogaster* genomic sequence) was replaced with a synthetic sequence retaining the wild type intron sequences and encoding the wild type Osk protein sequence, but with synonymous codons to maximize differences in the DNA sequence (see Supplementary File S1). *Drosophila* genomic sequence coordinates were obtained from FlyBase (Larkin *et al.* 2021).

Alleles of *osk, osk^0^*, *and osk^N^*, have been described (Kanke *et al.* 2015; Mohr *et al.* 2021). GAL4 drivers from the Bloomington Stock Center are (with abbreviations used here shown in parentheses) *P*(GAL4::VP16-nos.UTR)*CG6325[MVD1]* (*NGV*), BL#4937; *P*(matalpha4-GAL-VP16)*V37* (*MAT*), BL#7063; *P*(GAL4-nos.NGT)*40* (*NGT*), BL#4442. Additional GAL4 drivers from the Janelia Research Campus collection (a.k.a. the “Rubin GAL4 lines”), *12B02*, *25D06*, *29E01*, and *34C10*, were obtained from Allan Spradling. They were among a group that he identified when asked if his lab had tested, with positive results, any of the Janelia collection for activity in the ovary.

Egg laying assays were performed as described (Kenny *et al.* 2021).

### Detection of RNAs and proteins

RNA levels were determined by qPCR as described (Kenny *et al.* 2021). *In situ* hybridization to detect *osk* mRNA made use of tiled short DNA oligonucleotides (smFISH) 3*'*-end labeled with Quasar 670 fluorophore (LGC Biosearch Technologies) and used at 1.5 nM. Assays were performed as described (Abbaszadeh and Gavis 2016). Immunodetection of proteins in ovaries was as described (Ryu and Macdonald 2015), with a different fixation protocol for gamma-tubulin (Kenny *et al.* 2021). TO-PRO-3 Iodide (Invitrogen) (1:1000) or DAPI (1:4000) were used to stain DNA. Antibodies for imaging were mouse anti-1B1 which detects Adducin (Hts) protein (Developmental Studies Hybridoma Bank), diluted 1:1, mouse anti-Lamin Dm0 (ADL84.12)(Developmental Studies Hybridoma Bank), diluted 1:100, and rabbit anti-gamma-tubulin (Sigma T-0950), diluted 1:2000. Samples were imaged with a Nikon C2+ laser scanning confocal microscope. For all imaging experiments, the samples were obtained from at least five flies, typically many more. Quantification of *in situ* hybridization data was done in FIJI, with germaria or egg chambers traced and maximum pixel intensity measured. Measurements of oocyte areas bounded by gamma-tubulin foci were performed as described (Kenny *et al.* 2021).

### Statistical methods

Reproducibility was confirmed by performing independent experiments. Imaging experiments involved examination of multiple individual egg chambers in each experiment. Here, repetition served to reveal any technical problems, and the large number of individual egg chambers scored in each experiment ensured consistency and reproducibility. Egg laying experiments were performed at least three times. Rates of egg laying often show circadian variation, but because each experiment consisted of egg collections over several days, this source of variation was minimized. The experiments were not randomized, and no statistical method was used to predetermine sample size. One-way ANOVA was used to ask if there were significant differences among data sets with three or more variables. For all analyses Shapiro-Wilk tests rejected normality, and Wilcoxon rank-sum tests were used for *post hoc* analysis.

## Results and discussion

### Development and validation of new *osk* KD reagents

**Figure 1:**
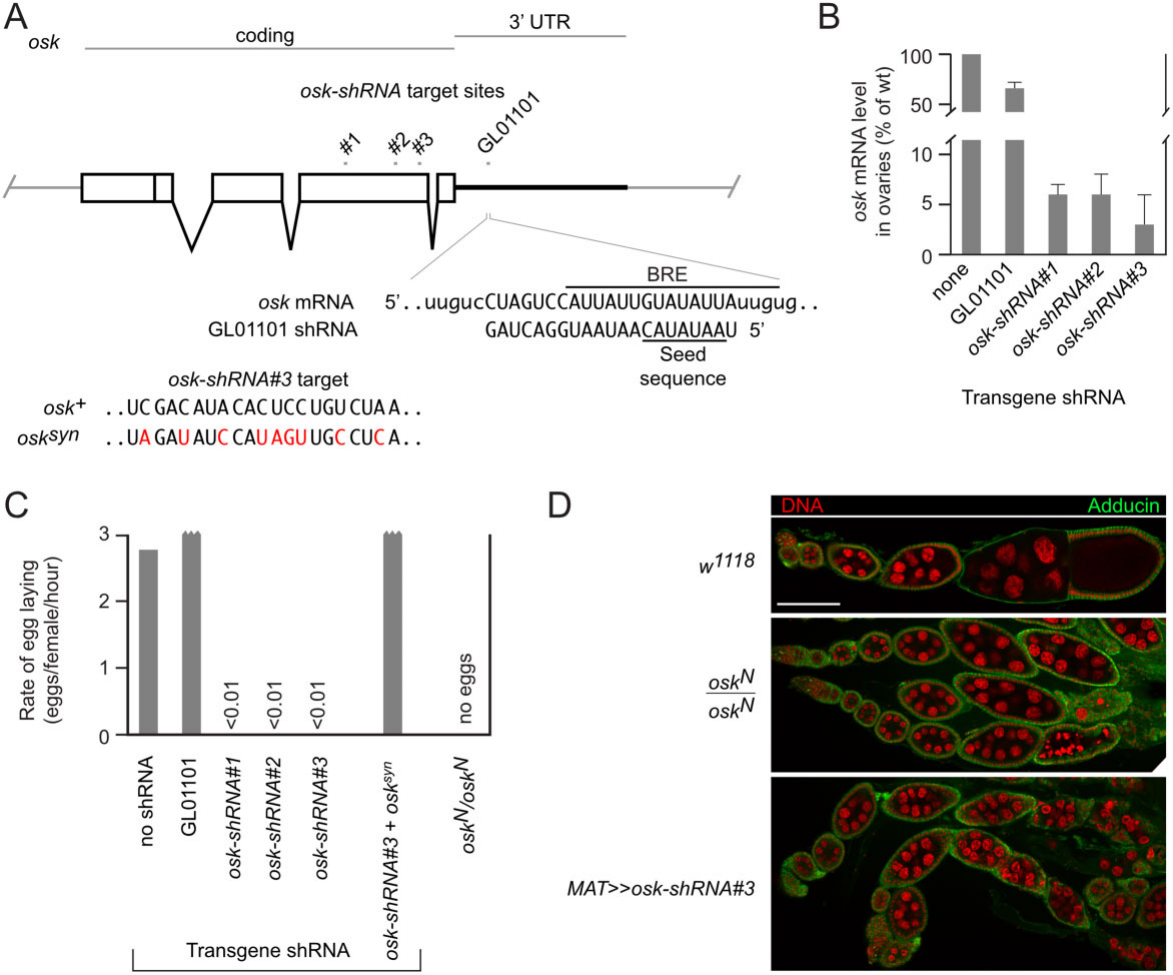
Knockdown of *osk* mRNA in oogenesis. (A) Diagram of the *osk* gene showing the positions of *osk-shRNA* target sites. RNA sequences directly below show the overlap between a Bru1 binding site (BRE) and the TRiP project *osk-shRNA* (GL01101) target. RNA sequences at bottom show the sequences mutated (red) in the *osk^syn^* transgene at the site of the *osk-shRNA#3* target, with nucleotides arranged by codon. (B) Levels of *osk* mRNA, assayed by qPCR, from ovaries of females in which the *MAT* GAL4 driver was present in combination with the *osk-shRNA* indicated. Error bars indicate standard deviations. (C) Rates of egg laying by females in which the *MAT* GAL4 driver was present in combination with the *osk-shRNA* indicated, except for the *osk^N^/osk^N^* genotype. For the sample tested in the presence of the *osk^syn^* transgene the *osk* background was *osk^N^/osk^+^* to compensate for the transgenic copy of *osk*. Values above 3 eggs/female/hour are truncated (jagged bar end): the *w^1118^* stock used as a wild type control often has a slightly lower rate of egg laying than various experimental strains. (D) Ovarioles showing the timing of oogenesis arrest from *osk* mutant and KD. Scale bar is 100 µm.

One approach to defining the critical phase or phases of *osk* ncRNA activity is to remove *osk* RNA by knock down (KD), relying on GAL4 drivers with different temporal activities to express a transgenic shRNA that targets *osk* mRNA. However, the maternal triple driver (*MTD-Gal4*) in combination with the *osk* shRNA transgenic reagent for germ-line expression (TRiP.GL01101) from the Transgenic RNAi Project (TRiP) is not effective at mimicking the most dramatic *osk* RNA null phenotype, arrest of oogenesis, as egg laying is not substantially reduced (Liu and Lasko 2015). Similarly, the *matalpha4-GAL-VP16* driver (henceforth referred to as *MAT*; abbreviations used for other drivers are given in MATERIALS AND METHODS) in combination with TRiP.GL01101 only modestly reduced the level of ovarian *osk* mRNA (Figure  1B) and failed to substantially reduce egg laying (Figure  1C).

Possible explanations for the weak KD include insufficient driver activity or limited efficacy of the shRNA during oogenesis. Notably, the *osk* shRNA target site overlaps substantially with a binding site for Bru1 protein (Figure  1A), which acts in translational regulation of *osk* mRNA (Kim-Ha *et al.* 1995; Reveal *et al.* 2010). Bru1 is present from the earliest stages of oogenesis (Webster *et al.* 1997), and when bound to the *osk* mRNA might interfere with binding of the shRNA, thus limiting the KD effect.

Three new transgenes for expression of shRNAs targeting other sites in *osk* mRNA were made and tested (*osk-shRNAs#1*, *#2*, and *#3*; Figure  1A). Using the *MAT* driver for expression, each new *osk-shRNA* dramatically reduced *osk* mRNA levels (Figure  1B) and largely eliminated egg production (Figure  1C). To confirm specificity for *osk* mRNA the KD with *osk-shRNA#3* was tested in the presence of an *osk* transgene—*osk^syn^—*with synonymous codon changes that alter the *osk-shRNA#3* target site (Figure  1A): egg laying was restored (Figure  1C). The *osk-shRNA#3* transgene was used for all subsequent *osk* KD experiments.

The contrasting properties of the original *osk-shRNA* and the novel *osk-shRNAs* strongly support the notion that Bru1 can disrupt binding of an shRNA when their targets in an mRNA are overlapping. It would not be surprising if a similar effect underlies a subset of other ineffective shRNAs, and compilations of shRNA effectiveness could potentially be mined for information contributing to the identification of recognition sites for other RNA binding proteins.

### Effectiveness of GAL4 drivers in reducing *osk* ncRNA activity

**Figure 2:**
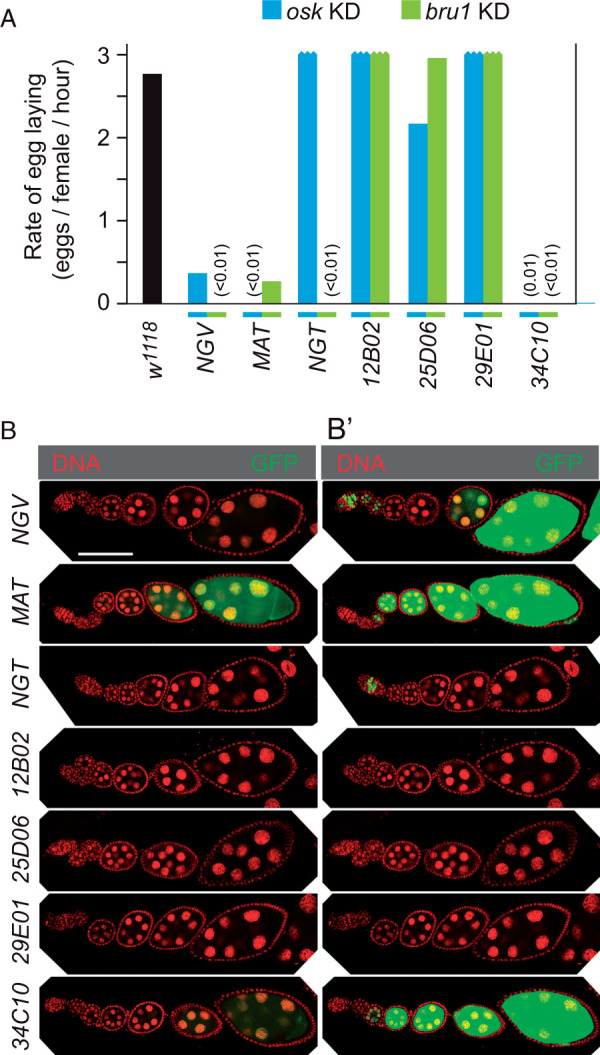
Comparison of GAL4 drivers for activity during oogenesis. (A) Rates of egg laying by females in which the indicated GAL4 driver was present in combination with transgenes for the KD of *osk* or *bru1*. (B) Expression patterns from the combination of *UAS-GFP* and the indicated GAL4 driver. Each panel shows a single ovariole from germarium (left) to stage 8 (right). Panel (B’) is the same set of panels with gain increased in the RGB green channel. Scale bar is 100 µm.

Several GAL4 drivers active in the female germ line were tested with *osk-shRNA#3* to monitor effects on *osk* ncRNA activity, focusing initially on egg laying (Figure  2A). For comparison, KDs of *bru1* were also performed. Females mutant for strong alleles of *bru1* produce no eggs and display an early arrest of oogenesis, the expected phenotype for an effective KD.

Three of the drivers tested, *NGV*, *MAT*, and *34C10*, substantially reduced egg laying in the *osk* KDs; each must be active at or before the stage when *osk* ncRNA activity is required for progression through oogenesis. When used for *bru1* KDs each of these drivers was also effective at arresting oogenesis. One driver, *NGT*, caused arrest of oogenesis in *bru1* KDs, but did not interfere with progression through oogenesis in *osk* KDs. Although the *osk and bru1* KDs are similar in causing arrest of oogenesis, the nature of the arrest is quite different. Whereas the *osk* KD allows progression through the early stages, the *bru1* KD arrest is associated with overproliferation of germ cells in pseudo egg chambers (Supplementary Figure S1), as observed in mutants lacking *bru1* activity (Parisi *et al.* 2001).

Each of these drivers was used in combination with a *UAS-GFP* transgene to compare periods of activity during oogenesis. Identical imaging settings were used for all genotypes. To detect lower levels of activity, the original images (Figure  2B) were adjusted identically in the green (GFP) channel to increase sensitivity (Figure  2B’). Only those drivers with substantial effects on egg laying in the *osk* KD were strongly active for *UAS-GFP* expression during the middle stages of oogenesis.

Both *MAT and 34C10* drivers produced GFP at substantial levels from stage 3 though at least stage 8 (the endpoint of this analysis). In principle, the continual appearance of GFP could arise from an initial phase of expression and persistence of the protein later in development, or from continual expression. Two observations suggest that persistence of GFP protein did not make a substantial contribution to the observed patterns. First, the half life of GFP appeared to be short relative to the time required for progression through oogenesis: GFP produced by the *NGT* driver in the germarium did not persist (Figure  2B’). Second, the intensity of the GFP signal from the *MAT and 34C10* drivers remained high and even increased from stages 3–8 (Figure  2, B and B’), despite a more than 10-fold increase in the volume of the egg chamber in that developmental period (King 1970). If there was only a short period of expression, the resulting GFP should have become progressively more dilute as volume increased.

### Consequences of removing *osk* ncRNA activity during the early stages of oogenesis

**Figure 3:**
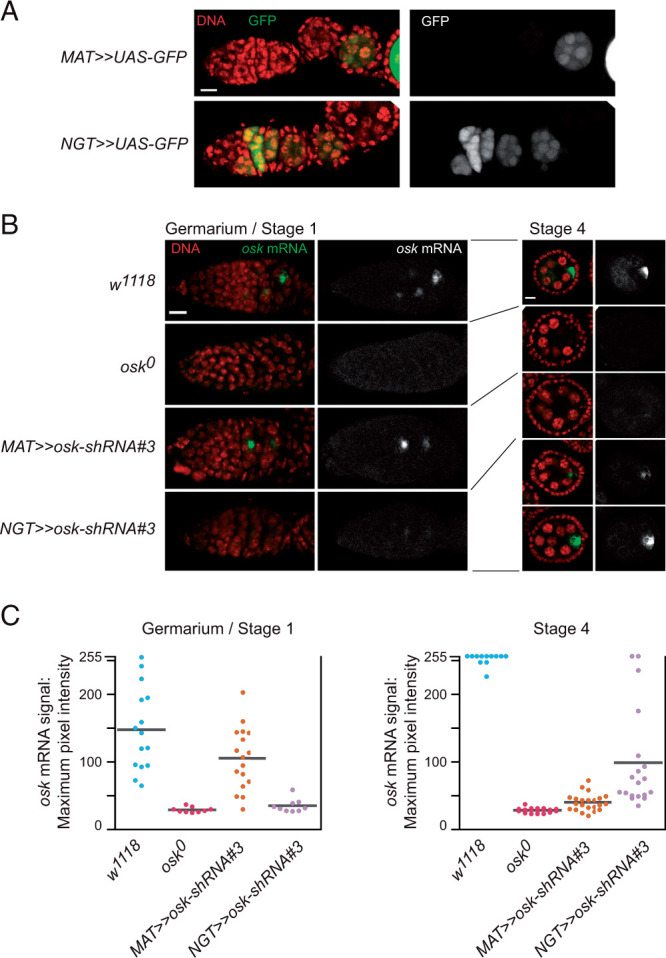
*osk* mRNA can be removed by KD early in oogenesis. (A) *MAT and NGT* driver activity in early oogenesis. Shown are the most anterior portions of ovarioles, with the germarium to the left and individual egg chambers to the right. Scale bar is 10 µm. Only the RGB green channel is shown in the panels at right. (B) *In situ* detection of *osk* mRNA at early stages of oogenesis. For both stages the *osk* mRNA signal from the left column is shown by itself in the right column. For stage 4 the levels of *osk* mRNA in the KD with the *NGT* driver were variable and two examples indicative of this variation are shown. Scale bars are 10 µm. (C) Quantitation of *osk* mRNA levels at early stages of oogenesis from *in situ* hybridization images (representative examples in panel B). For the stage 4 analysis imaging conditions were chosen to best reveal differences in low levels of *osk* mRNA in the *osk* mutant and KDs; consequently, there was signal saturation (pixel intensity of 255) for the wild-type sample. *P*-values were derived from the Wilcoxon rank-sum test. For all pairwise comparisons *P* < 0.01, with two exceptions: in the germarium/Stage 1 samples *P* < 0.1 for *w^1118^* vs *MAT≫osk-shRNA#3* and for *osk^0^* vs *NGT≫osk-shRNA#3*.

The *NGT* driver was active only very early in oogenesis: expression of the *UAS-GFP* reporter was detected in the germarium and in stage 2 egg chambers, in a developmental profile roughly complementary to *MAT* activity (Figures  2B’ and 3A). Despite the early activity of NGT, there was no effect on egg laying in the *osk* KD (Figure  2A). Thus, either NGT is not effective for the *osk* KD, or *osk* mRNA is not required at early stages for the progression through oogenesis. To address the effectiveness of the NGT KD of *osk*, we monitored *osk* mRNA levels by *in situ* hybridization. In the germarium, including stage 1 egg chambers not yet budded off, *osk* mRNA was largely eliminated (Figure  3, B and C). By stage 4 the *osk* mRNA levels had begun to recover: a substantial fraction of egg chambers still had very little *osk* mRNA while others had levels approaching that of the *w^1118^* control. By contrast, the *osk* KD with *MAT* caused only a modest reduction of *osk* mRNA in the germarium, while stage 4 egg chambers were substantially depleted (Figure  3, B and C). We conclude that *osk* ncRNA activity is not required during the earliest stages of oogenesis for progression through oogenesis. Moreover, the results with the NGT driver further narrow the definition of the critical phase for the requirement of *osk* ncRNA activity in progression through oogenesis. Because a significant fraction of stage 4 egg chambers in the *osk* KD with NGT still have extremely low levels of *osk* mRNA, yet there is no reduction in egg laying, *osk* ncRNA activity must be required only later. We conclude that at some point between stage 5 and the time of arrest at stage 6/7 *osk* mRNA performs the function that allows further progression through oogenesis.

Arrest of oogenesis is only one manifestation of absence of *osk* mRNA. Other *osk* RNA null phenotypes, such as altered organization of the MTOC (Kenny *et al.* 2021) and defective formation of the karyosome (Jenny *et al.* 2006; Kanke *et al.* 2015), can appear prior to the time of arrest. These processes are expected to be sensitive to loss of *osk* mRNA early in oogenesis from KD. Indeed, the distribution of *osk* mRNA, when present in stage 4 egg chambers after *osk* KD with *NGT* (Figure  3B), suggests an MTOC defect. In wild-type oocytes the MTOC from stages 2 to 6 is positioned at the posterior of the oocyte (Theurkauf *et al.* 1992), and this distribution underlies the posterior enrichment of multiple mRNAs including *osk* itself (Clegg *et al.* 1997). By comparison to wild-type stage 4 oocytes, in the *osk* KD with *NGT* the posterior enrichment of *osk* mRNA appeared to be reduced.

**Figure 4:**
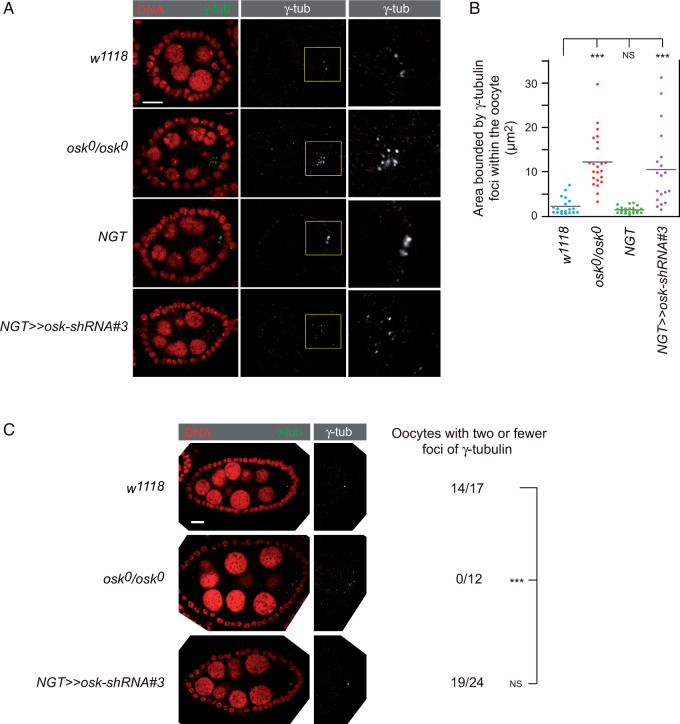
Disruption of MTOC from KD of *osk* in early oogenesis. (A) Representative stage 3-4 egg chambers from females with the genotypes indicated. Each egg chamber is oriented with posterior, the position of the oocyte, to the right. Left column: Complete egg chambers stained for gamma-tubulin (green) and DNA (red). Scale bar is 10 µm. Middle column: Images from the left column with only the gamma-tubulin signal (white). Right column: enlargements of the regions outlined in yellow in the middle column, containing the oocyte. (B) Measurements of the areas bounded by foci of gamma-tubulin in the oocyte (see Material and methods). At least 19 oocytes were analyzed for each genotype. The *P*-values were derived from the Wilcoxon rank-sum test: *** *P* < 0.01. (C) Representative stage 5–6 egg chambers from females with the genotypes indicated. Each egg chamber is oriented with posterior to the right. Left: complete egg chambers stained for gamma-tubulin (green) and DNA (red). Scale bar is 10 µm. The posterior portion with the oocyte is shown to the right with only the gamma-tubulin signal (white).

MTOC organization was monitored using gamma-tubulin as a marker, initially focusing on stage 3 and 4 egg chambers. Posterior enrichment is lost in the absence of *osk* mRNA, with multiple foci of gamma-tubulin present throughout the oocyte (Kenny *et al.* 2021) (Figure  4A). A similar defect was found in the early *osk* KD with the NGT driver (Figure  4, A and B).

The loss of *osk* mRNA from the *NGT* KD did not persist as oogenesis progressed. Consequently, we could ask if MTOC organization recovered as *osk* mRNA reappeared from ongoing transcription. In wild type stage 5 and 6 egg chambers, the clusters of gamma-tubulin transitioned into one or two predominant foci (Figure  4C). In the absence of *osk* mRNA, the abnormal distribution of gamma-tubulin persisted at later stages of oogenesis (Figure  4C). By contrast, in the early *osk* KD with the *NGT* driver the normal pattern was restored, with no significant difference from wild type by stages 5/6.

**Figure 5:**
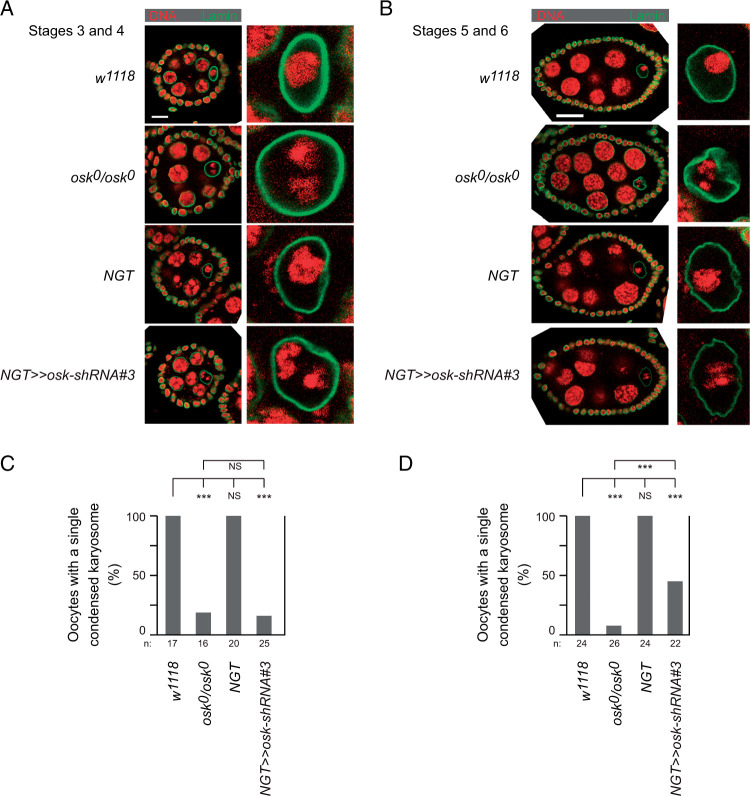
Disruption of karyosome formation from KD of *osk* in early oogenesis. (A) Representative stage 3–4 egg chambers from females with the genotypes indicated. Each egg chamber is oriented with posterior to the right. Left: complete egg chambers stained for DNA (red) and lamin (green) to outline the nuclei. Scale bar is 10 µm. Right: enlargements of the oocyte nuclei from the left images. (B) Representative stage 5–6 egg chambers from females with the genotypes indicated. Presented as in panel A, except that the scale bar is 20 µm. (C) Proportion of stage 3–4 oocytes of the indicated genotypes with normal karyosomes, defined as a single cluster of DNA in the oocyte nucleus. The number (n) of oocytes analyzed for each genotype is indicated. The *P*-values were derived from the Wilcoxon rank-sum test: *** *P* < 0.01. (D) Proportion of stage 5–6 oocytes of the indicated genotypes with normal karyosomes, defined as a single cluster of DNA in the oocyte nucleus. The *P*-values were derived from the Wilcoxon rank-sum test: *** *P* < 0.01.

We also monitored karyosome formation at the same early and mid stages of oogenesis. At stage 2 of oogenesis, chromosomes appear throughout the oocyte nucleus. With the completion of meiotic recombination the oocyte chromosomes condense to form the karyosome, a single compact cluster within the nucleus (King 1970) (Figure  5). Karyosome formation is defective in the absence of *osk* ncRNA activity, with the chromosomes partitioned into two or more foci (Jenny *et al.* 2006; Kanke *et al.* 2015) (Figure  5). KD of *osk* mRNA with the *NGT* driver produced the same defect (Figure  5A), statistically indistinguishable from the *osk^0^* mutant (Figure  5C).

The karyosome defects from the early *osk* KD with the NGT driver were substantially corrected as oogenesis proceeded and *osk* mRNA levels increased, although a significant fraction of egg chambers still had abnormal karyosomes at stages 5/6 (Figure  5, B and D; the example shown in Figure  5B is one in which the karyosome remains abnormal).

Because the initial failure to form the karyosome or to correctly position the MTOC did not prevent at least partial recovery when *osk* mRNA levels began to recover, the critical period for *osk* ncRNA activity in these two processes is ongoing and the role played by *osk* mRNA must occur repeatedly and not only once. The differing extents to which these defects were corrected could reflect different modes of action for *osk* ncRNA activity in each process, or different sensitivities to loss of *osk* mRNA. By either explanation, it seems unlikely that *osk* mRNA performs a single initial event, which then feeds into the different pathways.

## Data availability

The data underlying this article are available in the article and in its online supplementary material.

Supplementary material is available at *G3* online.

## Supplementary Material

jkab340_Supplementary_Figure_S1

jkab340_Supplementary_Figure
