# Supplementary figures and images for: Knock down analysis reveals critical phases for specific oskar noncoding RNA functions during Drosophila oogenesis
Source: G3 (Bethesda). 2021 Sep 29;11(12):jkab340. doi: 10.1093/g3journal/jkab340 (PMC8849117; doi:10.1093/g3journal/jkab340)

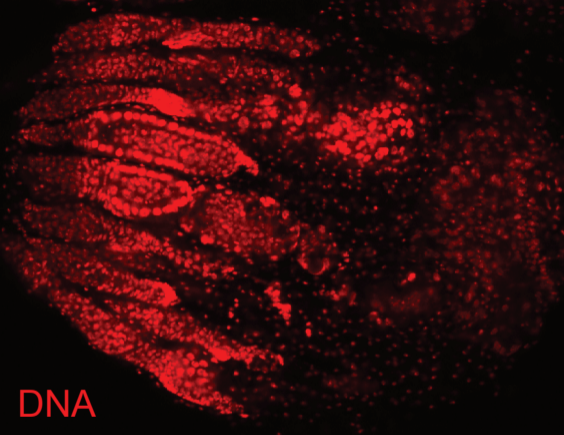

**Figure S1.** Phenotype of *bru1* KD with the *NGT* driver. A single ovary is shown with anterior to the left.

Supplement: jkab340_Supplementary_Figure_S1 [file jkab340_Supplementary_Figure_S1.pdf]
