# Supplementary material for: Knock down analysis reveals critical phases for specific oskar noncoding RNA functions during Drosophila oogenesis
Source: G3 (Bethesda). 2021 Sep 29;11(12):jkab340. doi: 10.1093/g3journal/jkab340 (PMC8849117; doi:10.1093/g3journal/jkab340)
Supplement: jkab340_Supplementary_Figure [file jkab340_Supplementary_Figure.rtf]

Sequence of the mutated region of osksyn. cTTtCTatTaACgATcCCtAAcGTcACgGCaGAaTGttcgGAaagCGGaAAaCGcATaTTtAACCTcAAgGCctcgCTCAAAAATGGCCATCTGCTCGACATGGTCCTGAACCAAAAGGAACGGACGTCGGATTACTCGTCGGGCGCACCCTCGCTCGAAAATATCCCGCGGGCTCCGCCTCGGTATTGGAAAAACCCGTTTAAGCGCAGAGCCCTCTCGCAACTCAACACGTCGCCCAGAACGGTCCCGAAAATCACCGACGAGAAAACGAAAGACATTGCGACGAGACCCGTATCCCTCCACCAGATGGCGAACGAAGCTGCCGAAAGCAATTGGTGTTATCAAGACAACTGGAAACATCTGTGAGTACTGAAAAGGTGTTTTGTAGACAGTGCAACTATAGCAAAAATAAACCTTTACTTAACAAAATGTACTAATCTTTCTACTGAACTCCTAGCAACAATTTCTATCAACAGGCGTCGGTGAACGCCCCGAAGATGCCGGTGCCGATTAATATTTACTCGCCGGACGCGCCGGAAGAGCCGATTAACTTAGCGCCGCCCGGCCACCAACCGTCCTGTAGGACGCAGTCTCAAAAGACGGAGCCCACGGAGAATCGGCACCTCGGAATTTTCGTCCACCCGTTCAATGGGATGAATATTATGAAACGCAGACATGAGATGACCCCGACCCCGACCATCCTCACGAGCGGCACGTATAATGACAGCCTCCTCACCATCAATTCCGACTATGACGCATACCTCCTCGATTTCCCACTGATGGGAGACGACTTCATGCTGTACCTGGCGCGTATGGAACTGAAGTGTAGGTTCAGAAGGCATGAACGCGTGCTCCAAAGTGGGCTCTGCGTGTCGGGCCTCACCATTAACGGGGCGCGTAACCGATTGAAGAGGGTGCAGTTGCCGGAAGGCACGCAAATTATTGTGAACATTGGCTCCGTCGATATCATGCGGGGGAAACCGCTCGTGCAAATTGAACATGACTTCCGCCTGCTAATTAAAGAAATGCATAACATGCGGCTCGTCCCAATCCTCACGAACCTCGCTCCCCTCGGGAATTACTGCCATGACAAAGTCCTGTGCGATAAGATTTATAGGTTTAATAAATTCATTCGGTCGGAGTGTTGCCATCTGAAAGTGATAGATATCCATAGTTGCCTCATTAATGAGCGCGGCGTCGTCCGCTTCGACTGTTTCCAGGCGTAAGACTTTATACCATATACCATATCATATGAGTTTACGATTTGTGCCACTTTTCAGCTCACCGCGGCAGGTGACGGGAAGCAAAGAGCCGTACCTCTTTTGGAACAAGATTGGCCGCCAACGGGTCCTCCAGGTCATCGAGACCTCCCTCGAATACTAA
